# External root resorption (ERR) and rapid maxillary expansion (RME) at post-retention stage: a comparison between tooth-borne and bone-borne RME

**DOI:** 10.1186/s40510-022-00439-y

**Published:** 2022-12-05

**Authors:** Rosalia Leonardi, Vincenzo Ronsivalle, Ersilia Barbato, Manuel Lagravère, Carlos Flores-Mir, Antonino Lo Giudice

**Affiliations:** 1 Department of General Surgery and Medical-Surgical Specialties, Section of Orthodontics, University of Catania, Catania, Italy; 2 Department of Oral and Maxillofacial Sciences, School of Dentistry, “Sapienza” University of Rome, Rome, Italy; 3 Orthodontic Graduate Program, University of Alberta, Edmonton, AB Canada

**Keywords:** RME, Tooth-borne RME, Bone-borne RME, ERR, Root resorption, Maxillary expansion, Orthodontics

## Abstract

**Background:**

The study aimed to compare external root resorption (ERR) three-dimensionally in subjects treated with tooth-borne (TB) versus bone-borne (BB) rapid maxillary expansion (RME). Forty subjects who received tooth-borne RME (TB group, average age 13.3 years ± 1.10 years) or bone-borne RME (BB group, average age 14.7 ± 1.15 years) were assessed using CBCT imaging before treatment (T0) and after a 6-month retention period (T1). 3D reconstructions of the radicular anatomy of maxillary first molars (M1), first and second premolars (P1 and P2) were generated to calculate volumetric (mean and percentage values) and shape changes (deviation analysis of the radicular models) obtained at each time point. 2D assessment of radicular length changes was also performed for each tooth. Data were statistically analyzed to perform intra-group (different teeth) and inter-group comparisons.

**Results:**

In both groups, all the investigated teeth showed a significant reduction in radicular volume and length (*p* < 0.05), with the first molars being the teeth most affected by the resorption process (volume and palatal root length). When volumetric radicular changes were calculated as a percentage of the pre-treatment volumes, no differences were found among the investigated teeth (*p* > 0.05). Based on the deviation analysis from radicular models superimposition, the areas most affected by shape change were the apex and bucco-medial root surface. Overall, the amount of ERR was significantly greater in the TB group (mm^3^: M1 = 17.03, P1 = 6.42, P2 = 5.26) compared to the BB group (mm^3^: M1 = 3.11, P1 = 1.04, P2 = 1.24).

**Conclusions:**

Despite the statistical significance, the difference in the amount of ERR of the posterior maxillary dentition between TB-RME and BB-RME remains clinically questionable.

**Supplementary Information:**

The online version contains supplementary material available at 10.1186/s40510-022-00439-y.

## Background

Transverse maxillary deficiency is a malocclusion seen among adolescents or adults with a prevalence of over 8–10% [1]. The treatment of this malocclusion is managed by applying strong forces (0.9–4.5 kg) on the maxilla midpalatal suture, through rapid maxillary expansion (RME) [2, 3], to increase the transverse widths of the maxilla. Tooth-borne (Hyrax expander) is the most frequently used RME appliance. Despite the benefits of RME, unwanted dento-alveolar side effects have been documented with tooth-borne expander, including external root resorption (ERR) [4–7]. In this regard, tissue-borne and bone-borne devices have been proposed with the assumption of reducing the application of forces to the posterior dentition [3, 6, 8].

Cone-beam computed tomography (CBCT) has proven a comparable accuracy to the Micro-CT method for ERR examination [9], with the latter methodology being limited to assessing extracted teeth due to its gantry size and excessive ionizing radiation [10]. Accordingly, some studies [10–13] have evaluated ERR following the application of RME, with the aid of CBCT. These studies showed a difference between pre-expansion and post-expansion root volumes since they found volume loss in the maxillary first molar (M1) and first molar (P1), and even in unattached second premolar (P2) [10, 12]. Moreover, using sophisticated engineering software and CBCT images, it is possible to generate 3D anatomical rendered models and analyze the anatomical surface changes (‘surface-to-surface’ analysis) by superimposition through a “best-fit” algorithm. A recent study [11] demonstrated significant surface area changes of M1 and P1 immediately after tooth-borne RME therapy.

However, almost all of the CBCT studies except for two [3, 10] examined only the immediate post-expansion amount of root resorption associated with tooth-borne and tissue-tooth-borne expanders. As the cementum is expected to undergo a degree of physiological repair, it would be more valuable to postpone the assessment of the radicular changes at the post-retention stage, to perform a comprehensive analysis of these natural repair mechanisms. Accordingly, the present study aimed to evaluate the radicular changes (volume, length, and surface data) of posterior maxillary teeth in patients treated with tooth-borne and bone-borne RME by analyzing CBCTs taken before expansion (T0) and after six months of retention (T1). The null hypothesis was that there was no difference in the extent of root resorption between TB and BB-RME during the post-retention period.

## Methods

The present study is the continuation of previously published studies involving the comparative analysis of skeletal and dento-alveolar effects between TB and BB expanders. Data were retrieved from the same sample of adolescents diagnosed with transverse skeletal deficiency and underwent CBCT examinations before treatment and after 6 months of retention [14–16]. The study was approved by the Health Research Ethics Board of Alberta University–Canada (protocol number: 00075765).

The sample consisted of 40 subjects (16 males, 24 females) with a mean age of 14.01 ± 1.29 years, respectively, divided into TB group (9 males, 11 female; mean age 13.3 ± 1.02 years) and BB group (7 males, 13 female; mean age 14.7 ± 1.15). Inclusion criteria: permanent dentition, completion of apexification of M1, P2, and P1, availability of adequate initial and post-retention records (good quality CBCT scans with a large field of view (FOV), photographs, dental casts, and medical history of each patient). Exclusion criteria: apical lesions and/or root canal treatment of the upper first molars and the first and second premolars, presence of any already diagnosed oral or systemic disease, prescribed medication, previous orthodontic treatment, maxillofacial surgery, or facial trauma. Information about the characteristics of the RME appliances, clinical protocol, and CBCT acquisitions have been previously reported [14–16].

**Fig. 1:**
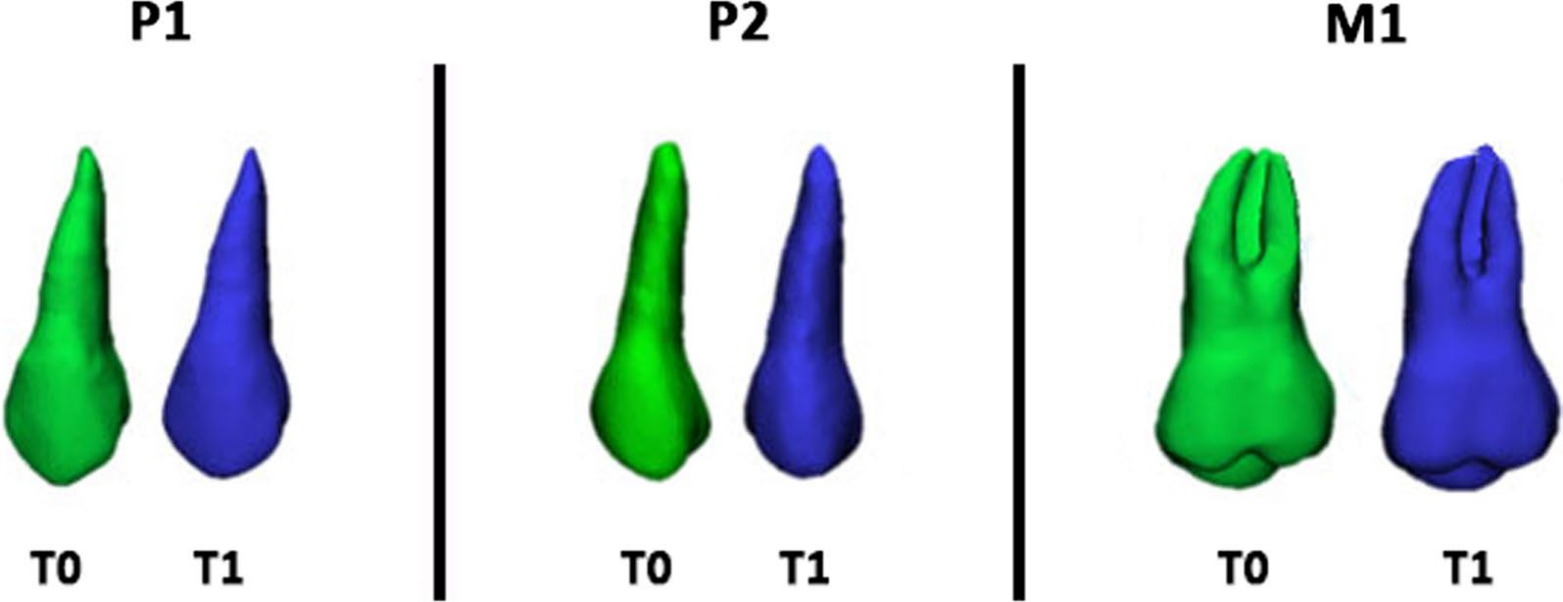
Color-coded labeling of T0 and T1 tooth models: maxillary first premolar (P1), maxillary second premolar (P2), and maxillary first molar (M1)
Step 2: *Root length measurements.* The original 3D models of each tooth were imported into 3-Matic Medical software (vr. 13.0, Materialise NV, Leuven, Belgium). The distances between these occlusal cusps and the most apical point on the radicular surface were measured (Fig. 2).

The method used in this study for the digital analysis of radicular changes consisted of 6 steps (Additional file 1: Fig. S1):

Step 1: *Segmentation process and 3D model rendering.* Segmentation masks of upper and lower first molars (M1), first premolars (P1), and second premolars (P2) were generated with Mimics Medical Software (Materialise NV vr.21.0, Leuven, Belgium) and color-coded to distinguish the time points for further analyses (Fig. 1). Lower dentition was also segmented to obtain data from a control sample.

**Fig. 2:**
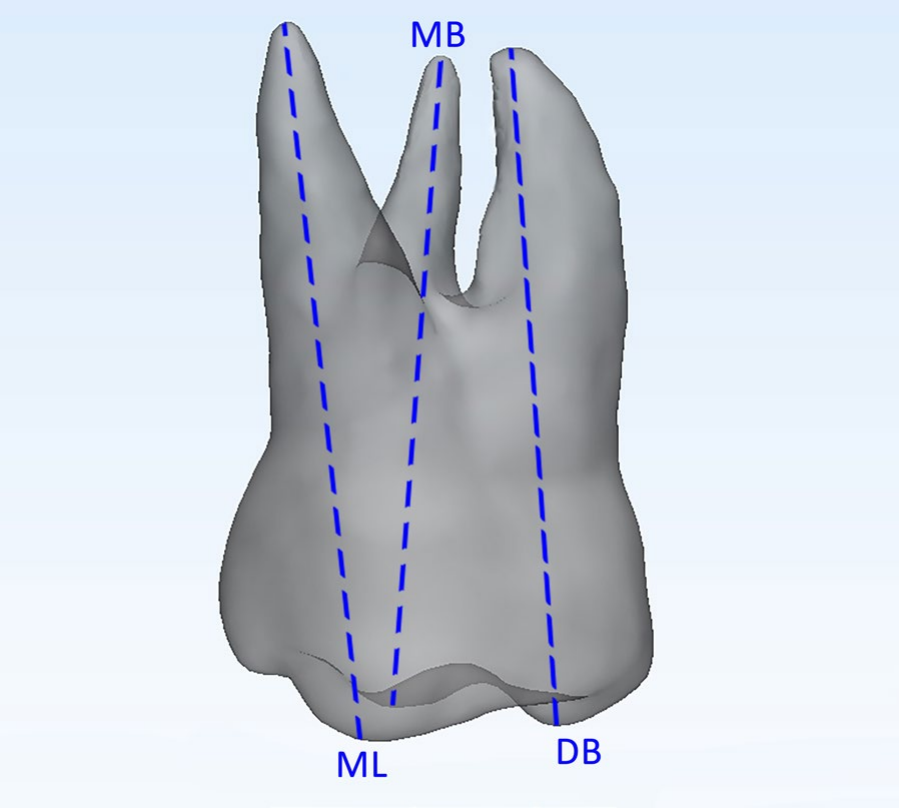
Assessment of root length. Mesiobuccal root assessed as the linear distance between the tip of the mesiobuccal cusp (MB) and the apex of the mesiobuccal root; distobuccal root assessed as the linear distance between the tip of the distobuccal cusp (DB) and the apex of the distobuccal root; and palatal root assessed as the linear distance between the tip of the mesiolingual cusp (ML) and the palatal root apex
Step 3: *3D radicular model template for 3D analysis.* In the Mimics software, the T0 mask of each tooth was duplicated. Two landmarks were placed on the duplicated model, respectively, on the lingual (CEJL) and buccal (CEJB) aspects of the crown at the cementoenamel junction level. An arbitrary plane passing through these points, and closed to the cementoenamel junction, was drawn. Afterward, the duplicated model was cut along this plane, and the radicular 3D model for each tooth was obtained (Fig. 3a–d). Finally, the final radicular templates were imported into 3-Matic Medical software.

**Fig. 3:**
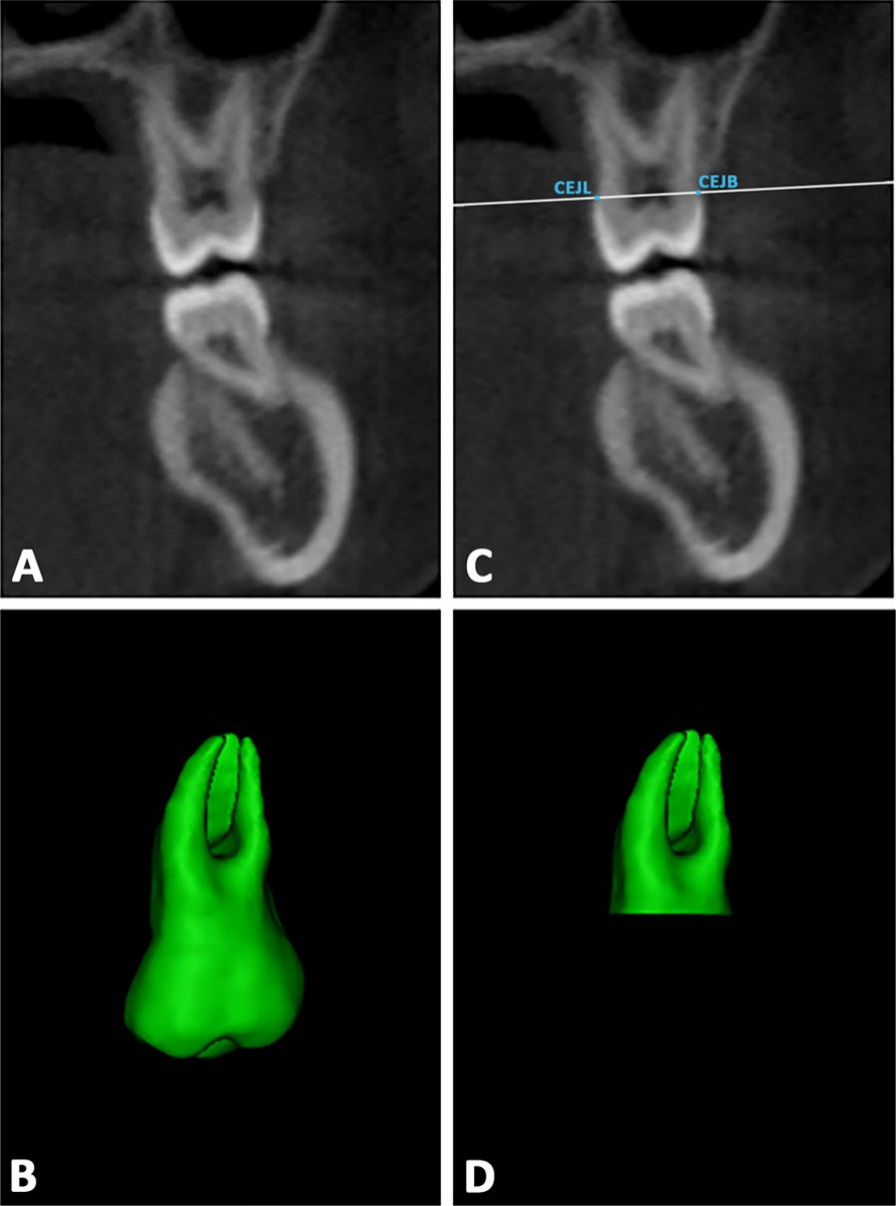
**a**–**b** Definition of the plane cut passing through two landmarks placed on the midpoint of palatal and buccal aspects of the crown at the cementoenamel junction (CEJ) level; **c**–**d** generation of the radicular 3D model template for each tooth investigated
Step 4: *First superimpositions (T0, T1 3D models) and surface-based registration.* In the 3-Matic software, a point-based superimposition between T0 and T1 original models was carried out (Fig. 4a, b) [11], followed by a global surface-based registration (best fit) of the 3D tooth models (Fig. 4c).

**Fig. 4:**
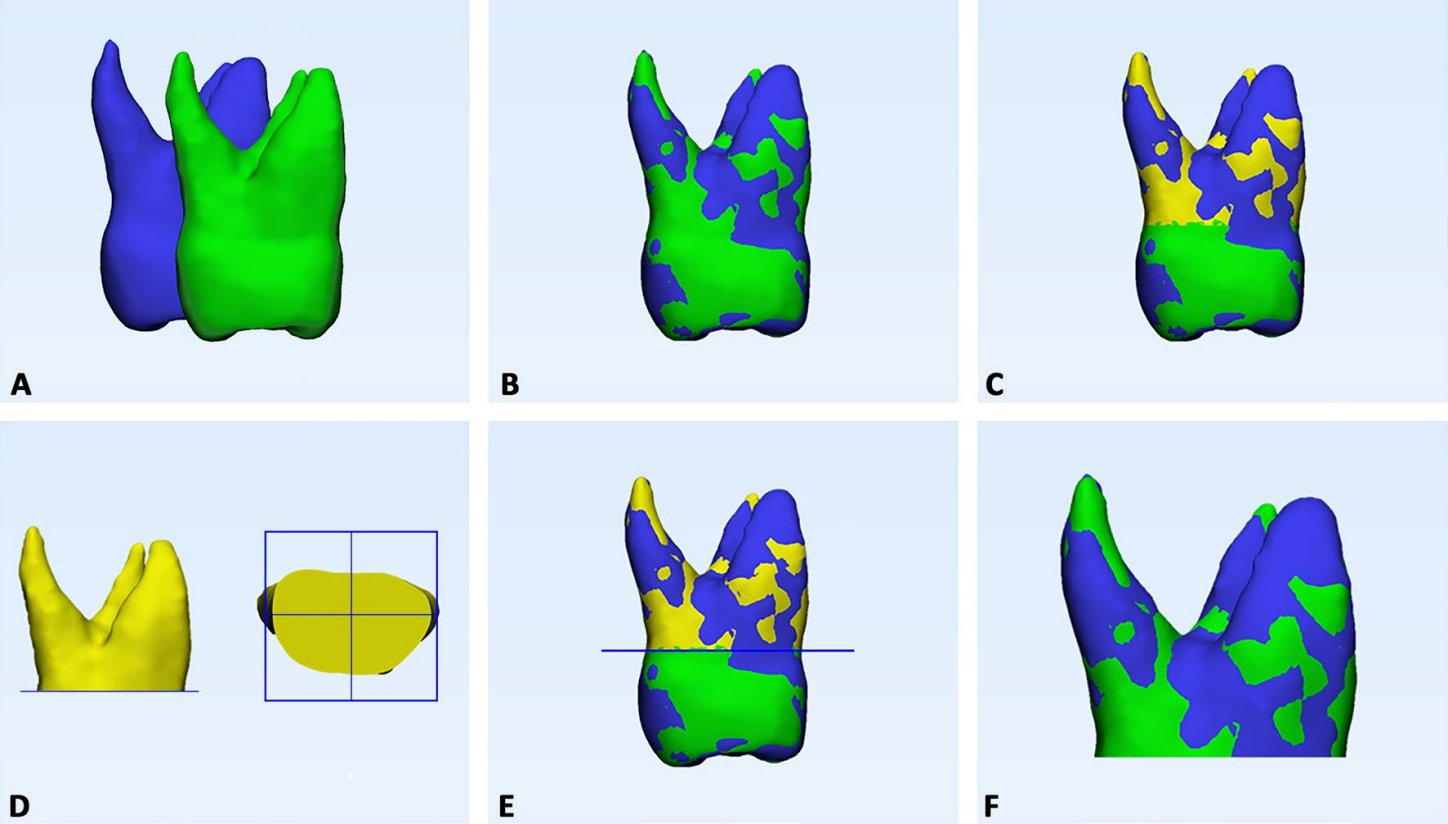
Registration of T0–T1 3D tooth models and crown removal from 3D models. **a** Preliminary point-based superimposition, by selecting five random points on the buccal, palatal/lingual, mesial approximal, distal approximal, and occlusal aspects of the two models of the same tooth. **b** Global registration using best-fit algorithm. **c** T0 tooth model (blue) and radicular template (yellow) and T1 tooth model in the same spatial orientation after superimposition; **d**, **e** definition of a single plane cut by randomly selecting three points on the lower surface of the T0 radicular template; **f** generation of the final T0 and T1 radicular models
Step 5: *Crown cut from 3D models.* Three points were randomly selected on the lower surface of the T0-3D radicular model to generate a plane cut to remove the crown from the teeth at the same level (Fig. 4d, e). Accordingly, the final T0 and T1 radicular models were obtained (Fig. 4f).
Step 6: *Volume measurement*, 3*D Deviation analysis and matching percentage calculation.* The radicular 3D models were imported into Geomagic Control X software (version 2017.0.0, 3D Systems, CA, USA) and radicular volumes were measured. Afterward, surface-based deviation analysis was carried out based on data from all points of the surface shells [14]. The values were represented on a color map according to the range of tolerance that was set a ± 0.3 mm (Fig. 5).

**Fig. 5:**
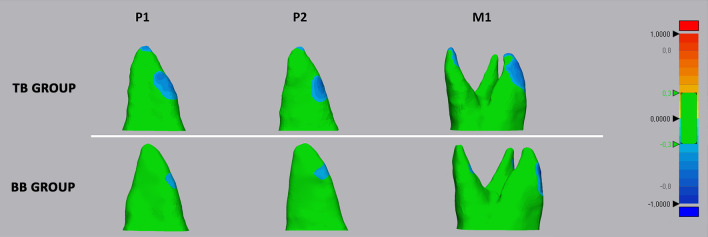
Deviation analysis between the T0 and T1 radicular models of first molar (M1), first premolar (P1), and second premolar (P2) in both tooth-borne (TB) and bone-borne (BB) expander groups. The colored map shows the deviations (negative blue, positive red) between the mesh models. The range of tolerance (green color) was set at ± 0.3 mm. The color-coded map showed that the reduction in cementum (blue-tone) was localized in the apical, bucco-apical, and bucco-medial radicular areas of both abutment and un-anchored teeth in the TB group. A similar resorption pattern was identified in the BB group, despite the absence of detectable deviation at the apex

### Statistical analysis

According to a preliminary analysis, the sample size was adequate to reach the 80% power to detect a mean difference of 10.61 mm^3^ between the radicular volumetric changes recorded in the post-retention phase (T0–T1) between P1 and M1, with a confidence level of 95% and a beta error level of 20%. The normal distribution and equality of variance of the data were performed with Shapiro–Wilk normality test and Levene’s test.

Chi-square test and Student’s *t* test were used, respectively, to assess the homogeneous distribution of gender, age, and skeletal expansion between TB and BB groups. The one-way analysis of variance (ANOVA) was used to evaluate the changes in radicular volumes, radicular lengths, and the matching percentage among the investigated teeth, and the Bonferroni test was used for post hoc comparisons. The unpaired Student’s *t* test was used to investigate the changes of radicular volume, radicular length and percentage of matching (T0-T1 superimpositions) between TB and BB groups for each tooth investigated. The same test was also used to perform a preliminary comparison between the right and left sides; since no differences were found, right and left teeth of the same type were pooled [11]. Intra-examiner reliability and inter-examiner reliability were assessed using the intraclass correlation coefficient (ICC). Data sets were analyzed using SPSS^®^ version 24 Statistics software (IBM Corporation, 1 New Orchard Road, Armonk, New York, USA).

## Results

The variables of gender, age, and obtained skeletal expansion (palatal width) were found to be equally distributed between both groups (data not shown). In both TB and BB groups, all the investigated teeth showed a significant volumetric reduction (*p* < 0.05) from T0 to T1, with M1 showing greater volumetric changes compared to P1 and P2 (*p* < 0.05) in both groups. Instead, no differences were found in the percentage of the total radicular volume among the investigated teeth (*p* > 0.05). The volumetric loss (mm and %) was significantly greater in the TB group compared to the BB group for each tooth investigated (*p* < 0.05) (Table 1).

**Table 1:** Descriptive and inferential statistics of radicular volumetric changes (mm3 and percentage) occurred after maxillary expansion

	T0–T1 (mm^3^)									T0–T1 (%)								
			TB group			BB group				TB group		BB group						
	Teeth	*n*	Mean	SD	*p* value*	Mean	SD	*p* value*	*p* value**	Teeth	*n*	Mean	SD	*p* value*	Mean	SD	*p* value*	*p* value**
Upper (Test)	P1	20	6.42	3.63	*p* < 0.05	1.04	0.62	*p* < 0.05	*p* < 0.05	*t*	20	5.39	2.75	NS	0.89	0.63	NS	*p* < 0.05
	P2	20	5.26	3.62		1.24	0.57		*p* < 0.05	*t*	20	4.05	2.6		1.02	0.48		*p* < 0.05
	M1	20	17.03	9.55		3.11	2.4		*p* < 0.05	*t*	20	5.35	2.94		0.99	0.79		*p* < 0.05

*P1* first premolar; *P2* second premolar; *M1* first molar; *TB* tooth borne; *BB* bone borne; *T0* pre-treatment; *T1* post-retention; *n* = number of teeth; *SD* standard deviation; *NS* not significant

*p* value* based on one-way analysis of variance (ANOVA) for intra-group comparison (different teeth) and set at *p* < 0.05; post hoc assessment performed according to the Bonferroni's multiple comparisons test

*p* value** based on independent Student’s *t* test for inter-group comparison and set at *p* < 0.05

In both TB and BB groups, all the investigated teeth showed a significant reduction in the radicular length from T0 to T1 (*p* < 0.05), with M1p root mostly affected by length reduction (*p* < 0.05). The changes in radicular length were significantly greater in the TB group compared to BB group for each tooth investigated (*p* < 0.05) (Table 2).

**Table 2:** Descriptive and inferential statistics of radicular length changes occurred after maxillary expansion

T0–T1 (mm)								
		TB group			BB group			
Teeth	*n*	Mean	SD	*p* value*	Mean	SD	*p* value*	*p* value**
P1	20	0.31	0.16	*p* < 0.05	0.04	0.02	*p* < 0.05	*p* < 0.05
P2	20	0.23	0.12		0.07	0.03		*p* < 0.05
M1m (c)	20	0.22	0.10		0.07	0.03		*p* < 0.05
M1d (d)	20	0.25	0.15		0.06	0.02		*p* < 0.05
M1p (e)	20	0.33	0.19		0.11	0.04		*p* < 0.05

*P1* first premolar; *P2* second premolar; *M1m* first molar mesial root; *M1d* first molar distal root; *M1p* first molar palatal root; *TB* tooth borne; *BB* bone borne; *T0* pre-treatment; *T1* post-retention; *n* number of teeth; *SD* standard deviation

*p* value* based on one-way analysis of variance (ANOVA) for intra-group comparison (different teeth) and set at *p* < 0.05; post hoc assessment performed according to the Bonferroni’s multiple comparisons test

*p* value** base on independent Student’s *t* test for inter-group comparisons and set at *p* < 0.05

In both TB and BB groups, significant differences in the percentage of matching were found among P1, P2, and M1 (*p* < 0.05), when superimposing T0 to T1 3D models. The M1 showed a limited percentage of matching compared to P1 and P2. All the investigated teeth showed a significantly higher percentage of matching in the TB group (*p* < 0.05) (Table 3).

**Table 3:** Comparison of matching percentage of pre-treatment and post-retention radicular shells (T0–T1 superimposition) for each tooth investigated

		T0–T1 matching (%)						
		TB group			BB group			
Teeth	*n*	*Mean*	*SD*	*p* value*	*Mean*	*SD*	*p* value*	*p* value**
P1 (a)	20	92.83 (c)	2.65	*p* < 0.05	96.51 (c)	2.53	*p* < 0.05	*p* < 0.05
P2 (b)	20	93.27 (c)	3.11		97.8 (c)	2.01		*p* < 0.05
M1 (c)	20	89.33 (a, b)	4.51		93.04 (a, b)	3.40		*p* < 0.05

*P1* first premolar; *P2* second premolar; *M1* first molar; *n* number of teeth; *SD* standard deviation

*p* value* based on one-way analysis of Variance (ANOVA) for intra-group comparisons (different teeth) and set at *p* < 0.05

*p* value** for inter-group comparisons, based on independent Student’s *t* test and set at *p* < 0.05

No differences were found in the control sample (lower teeth) for all the parameters investigated between T0 and T1 (*p* > 0.05), with mean difference values close to 0 (data not shown).

Correlation indexes for intra-operator readings ranged from 0.915 to 0.934 for radicular volume assessments and from 0.924 to 0.953 for linear measurements; correlation indexes for inter-operator readings ranged from 0.867 to 0.893 for radicular volume assessments and from 0.909 to 0.942 for linear measurements.

## Discussion

The present findings would confirm that RME reduces the radicular volume of the maxillary first molars, first and second premolars in the post-retention stage (T0-T1). The first molars showed consistently greater values of volume loss; however, when this parameter was calculated as a percentage of total radicular volume, no significant differences were identified among the investigated teeth. In the tooth-borne RME, this would mean that posterior teeth could be exposed equally to ERR, whether they act as abutment teeth (P1 and M1 in this study) or as un-anchored teeth (P2), that is, even if they received different loads, as also suggested by previous evidence [12]. The values of volumetric loss detected in the TB group were similar to those reported by an earlier study testing conventional maxillary expanders [10]. Considering that the age of the study sample (TB = 13.3 ± 1.02; BB = 14.7 ± 1.15) approximates nearly the final maturational stage of the premolars [17], it could be assumed that the ERR detected may have disrupted the final developmental stage of these teeth.

We also assessed radicular length changes and the shape between the radicular T0 and T1 3D models. All investigated teeth reported a reduction in radicular length in the post-retention stage, with M1p and P1 being the roots mostly involved in the TB group (respectively 0.33 mm and 0.31 mm of length reduction), and with M1p being the root mostly involved in the BB group (0.11 mm of length reduction). In this regard, one of the main concerns of ERR is the harmful consequence of root shortening on the tooth longevity; however, the values registered in this study should be far from threatening the function of the dentition significantly, at least from a quantitative perspective, considering that 2-mm root shortening was found to reduce the total attachment area of 5–10% [18, 19]. In summary, these differences are likely to be considered clinically irrelevant.

The color-coded map obtained from T0 to T1 deviation analysis showed that the reduction in cementum (showed by blue-tone) was localized in the apical, bucco-apical, and bucco-medial radicular areas of both abutment and un-anchored teeth in the TB group. A similar resorption pattern was identified in the BB group, despite the absence of detectable deviation at the apex. This would suggest that modifications in this region were likely irrelevant, as confirmed by the linear measurements of radicular length. These findings corroborate previous evidence from histological materials reporting the generation of radicular resorption on the buccal surface of the roots in the form of small irregularly shaped lacunae [5, 8], and from recent well-conducted micro-CT studies [3]. In this regard, it robustly supports such patterns of ERR: The forces generated by RME are orientated toward the buccal side of the dento-alveolar arch, causing the compression of the periodontal ligament and subsequent hyalinization on the buccal side of the roots, and ERR occurs during the elimination of the hyalinization tissue on the compressed side [20]. Furthermore, the root apex could be a sensitive area since greater force per unit of the surface area generated during RME and because of the presence of a thicker and more rigid bone compared to the trabecular bony architecture of the cervical region [21].

A recent CBCT study [10] showed that maxillary premolars and first molars featured a slight recovery of radicular volume between the active and post-retention phases of RME. This recovery would reflect the process of repair of the damaged cementum. It has been seen that when the orthodontic forces ceased or are below a certain level, the removal of the hyalinized necrotic tissue begins with the subsequent cemental repair [20, 22–24]. Thus, it may be possible that the amount of root resorption detected in the present investigation could have been influenced by an active process of cementum repair that might have mitigated the damage that occurred after the active expansion phase.

The null hypothesis of the present study is rejected since the amount of root resorption recorded was significantly greater in TB group compared to the BB group. These findings are explained since no direct forces were applied to the dentition in the BB group, supporting the evidence from a recent split-mouth study [3]. Conversely, our findings disagree with another recent study where the authors have limited the observation to 2D linear measurements of radicular length [25]. However, it must be emphasized that the differences found in the present study between TB and BB expanders are not likely of clinical relevance, and should not influence the clinical decision of the anchorage system to use.

The fact that some amount of volume loss and length reduction was detected in the BB group may be related to the design of skeletal anchorage, which consists of two mini-screws placed on the palatal slope between the second premolar and the first molar area, concentrating the force on the posterior region that may have been somehow transmitted to the dentition due to its potential proximity [26]. In this regard, further studies testing different skeletal anchorage designs and using a consistent methodology for the evaluation of ERR are warmly recommended to provide more conclusive evidence.

## Limitations

- There are opposing opinions on the adequate spatial resolution of CBCT examinations for assessing radicular volume. In this regard, although it was found that ERR could be underestimated with voxel sizes greater than 0.2 mm [27], a more recent study found that there are no significant differences in sensitivity and specificity between 0.3 mm voxel size (used in the present study) and 0.15, 0.20, and 0.25 mm voxel sizes, but with the advantage of lower ionizing radiation exposure [28]. Also, the methodology applied in the present study relied on a specific CBCT machine (iCAT, Imaging Sciences International, Hartfield, PA). Consequently, the reliability of the segmentation process cannot be extrapolated to other CBCT machines since many CBCT systems do not provide readouts in Hounsfield units (HU) [29].
- Since the only clinical parameter used for standardizing the expansion protocol was the achievement of the overcorrection of the malocclusion, the presented findings of RR may be biased from the different amounts of palatal expansion required among individuals.

## Conclusions

- A significantly greater amount of ERR, assessed as volumetric reduction (3D analysis) and root shortening (2D analysis), was observed with tooth-borne RME. The magnitude of the differences could be considered clinically questionable.
- According to the deviation analysis, the ERR was primarily detected in the apical, bucco-apical, and bucco-medial radicular areas of 3D radicular models, which was coherent with the direction of the forces generated during RME.
- Even unattached teeth were affected by ERR, suggesting that the transmission of the forces could not be limited to the anchorage teeth.

## Supplementary Information

**Additional file 1: Fig. S1.** Flowchart of the digital work-flow involved in the present study

## Data Availability

The datasets used and analyzed during the current study are available from the corresponding author on reasonable request.
