# Supplementary figures and images for: External root resorption (ERR) and rapid maxillary expansion (RME) at post-retention stage: a comparison between tooth-borne and bone-borne RME
Source: Prog Orthod. 2022 Dec 5;23:45. doi: 10.1186/s40510-022-00439-y (PMC9719874; doi:10.1186/s40510-022-00439-y)

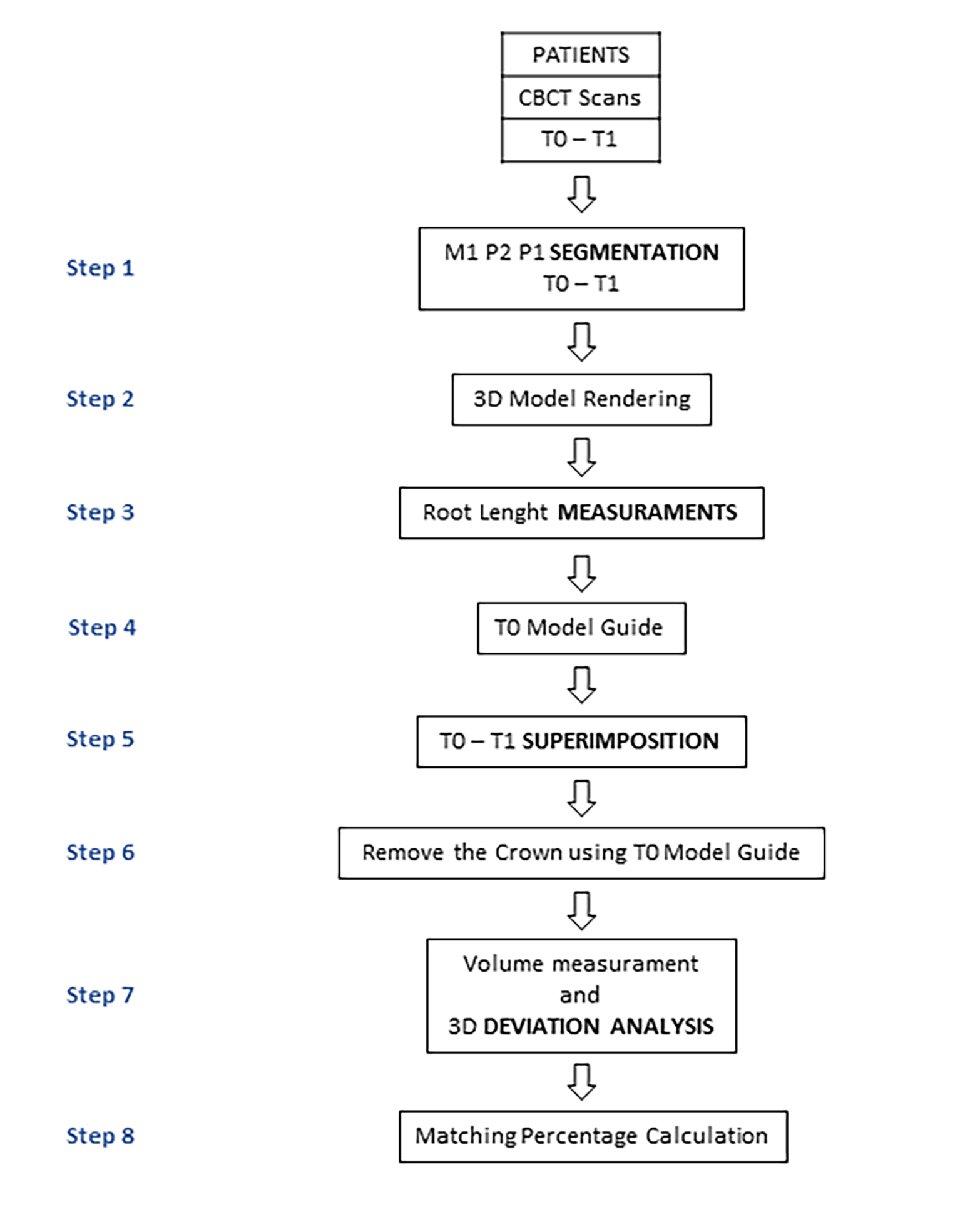

Supplement: Supplementary file 1 — Additional file 1: Fig. S1. Flowchart of the digital work-flow involved in the present study [file 40510_2022_439_MOESM1_ESM.tif]
